# Lactoferrin Contributes a Renoprotective Effect in Acute Kidney Injury and Early Renal Fibrosis

**DOI:** 10.3390/pharmaceutics12050434

**Published:** 2020-05-08

**Authors:** Yung-Ho Hsu, I-Jen Chiu, Yuh-Feng Lin, Yi-Jie Chen, Yu-Hsuan Lee, Hui-Wen Chiu

**Affiliations:** 1 Division of Nephrology, Department of Internal Medicine, Shuang Ho Hospital, Taipei Medical University, New Taipei City 23561, Taiwan; yhhsu@s.tmu.edu.tw (Y.-H.H.); d118105006@tmu.edu.tw (I.-J.C.); linyf@s.tmu.edu.tw (Y.-F.L.); 2 Department of Internal Medicine, School of Medicine, College of Medicine, Taipei Medical University, Taipei 11031, Taiwan; 3 Graduate Institute of Clinical Medicine, College of Medicine, Taipei Medical University, Taipei 11031, Taiwan; chenyj1108@tmu.edu.tw; 4 Department of Cosmeceutics, China Medical University, Taichung 40402, Taiwan; yhlee@mail.cmu.edu.tw

**Keywords:** lactoferrin, acute kidney injury, chronic kidney disease, autophagy, fibrosis

## Abstract

Patients with acute kidney injury (AKI) who survive the acute stage are at notable risk for chronic kidney disease (CKD) progression. There is no single therapy that can effectively prevent the AKI to CKD transition. Autophagy is a cytoplasmic component degradation pathway and has complex functions in several diseases, such as renal fibrosis. Previous research has shown that lactoferrin has important functions in antioxidant defense and other defense systems, protecting kidneys against various injuries. The present study investigated the effect of lactoferrin in protecting against the AKI to CKD transition. We identified 62 consensus genes with two-fold changes in clinical kidney tissues from AKI and CKD patients. Among the 62 overlay genes, the mRNA levels of LTF were significantly upregulated in the kidney tissues of AKI and CKD patients. Lactoferrin induced autophagy via the activation of the AMPK and inhibition of Akt/mTOR pathway in human kidney proximal tubular cells. Lactoferrin suppressed oxidative stress-induced cell death and apoptosis by augmenting autophagy. Lactoferrin has an antifibrotic role in human kidney tubular cells. In a mouse model of folic acid-induced AKI to CKD transition, treatment with lactoferrin recovered renal function and further suppressed renal fibrosis through the inhibition of apoptosis and the induction of autophagy. These findings identify lactoferrin as a potential therapeutic target for the prevention of the AKI to CKD transition.

## 1. Introduction

Acute kidney injury (AKI) is a critical illness that is related to increased morbidity and mortality [1]. Patients with partial AKI develop progressive and persistently aggravated proteinuria and decreased glomerular filtration rate (GFR) [2]. Previous studies have demonstrated that the recovery of renal function in patients with AKI is often incomplete. Furthermore, AKI is a critical risk factor for the development of chronic kidney disease (CKD) and end-stage renal disease (ESRD) [3,4]. AKI increases the risk of CKD by 8.8-fold and the risk for ESRD or kidney transplantation by 3.3-fold [3]. At present, research on the related mechanisms of AKI progression to CKD has mainly focused on persistent inflammatory response, mitochondrial dysfunction, microvascular endothelial cell injury and the abnormal activation of renal tubular epithelial cells. Moreover, AKI can often cause fibrous tissue hyperplasia, the release of fibrogenic factors and renal fibrosis [5,6]. Inflammation and oxidative stress are closely linked, as they generate a bad cycle. Oxidative stress starts inflammation. Inflammation, in turn, induces oxidative stress through the production of reactive oxygen species (ROS). These damaging events cause tissue injury by inducing necrosis, apoptosis and fibrosis [7]. Therefore, novel drugs or mechanisms that antifibrotic functions and accelerate fibrogenesis in treating the AKI to CKD transition must be developed.

Many recent studies have shown that autophagy plays an important role in several diseases, including kidney disease [8,9,10,11]. Autophagy is an intracellular degradation process that removes and recycles proteins and damaged organelles to maintain cellular homeostasis [9]. A previous study demonstrated that ATG5-mediated autophagy in tubules hampered the activation of NF-κB signaling to protect against renal inflammation [12]. Furthermore, autophagy attenuated G2/M cell cycle arrest in proximal tubular epithelial cells and renal fibrosis [13]. Our recent study indicated that resveratrol-loaded nanoparticles can be as a strategy to prevent CKD through the autophagy induction and NLRP3 inflammasome attenuation [14]. Another recent study concluded that overexpression of the SIRT6 gene inhibited apoptosis and induced autophagy, which might be involved in repairing kidney damage in AKI caused by sepsis [15]. However, whether autophagy plays a key role in the process of the AKI to CKD transition is still unknown.

Lactoferrin is a natural iron-binding glycoprotein originally isolated from milk. Lactoferrin is found notably in milk, mucosal secretions and other bodily fluids [16]. Previous research has shown that lactoferrin has multipharmacological properties, including antiviral, antibacterial, anti-inflammatory, and antioxidant properties [17,18,19,20]. In a screening for lactoferrin expression in various organs, kidneys were found to have high levels of lactoferrin mRNA and protein. This indicated that lactoferrin may have important functions in the antioxidant defense and other defense systems protecting kidneys against any other stresses [21]. In the current study, we found that the mRNA level of lactoferrin was elevated in the renal tissue of AKI and CKD patients compared to healthy individuals. The protective effect of lactoferrin has been assessed in a folic acid-induced AKI to CKD mouse model. We also investigated the roles of autophagy, apoptosis and fibrosis in lactoferrin-treated human kidney cells.

## 2. Material and Methods

### 2.1. Microarray Analysis

The microarray datasets (GSE66494 and GSE30718) were downloaded from the Gene Expression Omnibus (GEO) database. The raw data were normalized with GeneSpring software. The differential transcriptional activity of AKI patients and CKD patients was compared with healthy individuals. The results are shown as a boxplot and was produced using SPSS software.

### 2.2. Cell Culture and Lactoferrin Treatment

The human kidney proximal tubular epithelial cell line HK-2 was purchased from American type culture collection (CRL2190). HK-2 cells were cultured in keratinocyte serum-free (KCSF) medium with 40 μg/mL bovine pituitary extract and 5 ng/mL recombinant epidermal growth factor (Gibco BRL, Grand Island, NY, USA) at 5% CO_2_ and at 37 °C. For exposure to lactoferrin (Wako Pure Chemical Industries, Ltd., Osaka, Japan), 40 mg/mL fresh stock solutions were prepared. The solution was added to the culture medium and mixed.

### 2.3. Cell Viability Assay

We used sulforhodamine B (SRB) assay to detect cell viability. Briefly, the cells were fixed with a trichloroacetic acid solution for 1 h and then SRB (Sigma-Aldrich Corp. St Louis, MO, USA) was added for 1 h. After washing, 20 mM of Tris buffer was added. Finally, the absorbance at 562 nm was read by ELISA reader (Molecular Devices, Sunnyvale, CA, USA). The reference value for calculating 100% cell viability is the mean absorbance of the untreated cells.

### 2.4. Western Blot Analysis

Total protein was prepared from cell lysates using protein extraction buffer. The proteins at 30 μg/lane or TD-PM10315 TOOLS Pre-Stained Protein Marker (10–315 kDa) (BIOTOOLS, New Taipei City, Taiwan) were loaded on a SDS gel, subjected to electrophoresis, blotted, probed using antibodies and detected by a chemiluminescence (ECL) detection system (Thermo Fisher Scientific, Waltham, MA, USA). Anti-Akt, anti-p-Akt, anti-Beclin 1, anti-mTOR, anti-p-mTOR, anti-AMPK, anti-p-AMPK, anti-GAPDH, anti-caspase 3, anti-caspase 9, anti-LC3 and anti-PAI-1 antibodies were purchased from Cell Signaling Technology (Ipswich, MA, USA); anti-lactoferrin antibody was purchased from Biovision Inc. (Mountain View, CA, USA); and anti-Bax, anti-collagen and anti-CTGF antibodies were purchased from Proteintech Group (Chicago, IL, USA).

### 2.5. Immunofluorescence Microscopy

HK-2 cells were cultured on coverslips. After incubation, the cells were fixed in paraformaldehyde (4%) and blocked with BSA (1%) for 30 min. Then, the cells were incubated with an anti-LC3 antibody (MBL, Japan) for 1 h. The cells were washed in PBS and added with DyLight™ 488-conjugated AffiniPure goat anti-rabbit IgG (Jackson ImmunoResearch Laboratories, PA, USA) for 1 h and stained with 4’,6-diamidino-2-phenylindole (DAPI) (Invitrogen, Carlsbad, CA, USA). Then, the cells were washed in PBS and analyzed with a confocal microscope (Leica TCS SP5, Mannheim, Germany).

### 2.6. Transfection of siRNA and LTF DNA

The *LTF* plasmid was constructed by ligating *LTF* cDNA sequence from pCMV6-XL5-LTF (OriGene Technologies Inc., Rockville, MD, USA). Then, 2 μg *LTF* DNA was transfected into HK-2 cells in 6-well plate by Lipofectamine^®^ 2000 Reagent (Invitrogen, Carlsbad, CA, USA), according to the manufacturer’s instruction. The transfection of siRNA was utilized by TransIT-X2^®^ Dynamic Delivery System (Mirus, Madison, WI, USA) according to the manufacturer’s instruction. Beclin 1 siRNA (ID: s16539) was purchased from ThermoFisher Scientific (Waltham, MA, USA). Briefly, Opti-MEM I reduced-serum medium, siRNA solution and TransIT-X2 were mixed gently. The mixed solution was incubated at room temperature for 30 min to form complexes. Then, the complexes were added to cells for 24–72 h.

### 2.7. Folic Acid Mouse Model

Male C57BL/6 mice (eight weeks old) were purchased from the National Laboratory Animal Center (Taipei, Taiwan). The animals were housed five per cage with 50% ± 10% relative humidity at 24 ± 2 °C. The animals were acclimatized for 1 week prior to the start of experiments and fed a Purina chow diet with water ad libitum. The animal protocol was reviewed and approved by the Institutional Animal Care and Use Committee of Taipei Medical University, Taiwan (approval number: LAC-2018-0362). All animal experiments took place at Laboratory Animal Center of Taipei Medical University. The mice were divided into the following four groups (five mice/group): (1) equivalent volumes of saline administered intraperitoneally (i.p.) two times per week for 5 weeks starting at day 2 (normal group); (2) mice i.p. injected with 250 mg/kg folic acid one time at day 0 (Sigma-Aldrich) (FA group); (3) mice i.p. injected with 250 mg/kg folic acid one time at day 0 and i.p. injected with low-concentration (2 mg/mouse) lactoferrin two times per week for 5 weeks at starting day 2 (FA + LFL group); and (4) mice i.p. injected with 250 mg/kg folic acid one time at day 0 and i.p. injected with high-concentration (4 mg/mouse) lactoferrin two times per week for 5 weeks, starting at day 2 (FA + LFH group). The mice were sacrificed by CO_2_ exposure, and the kidney tissues were fixed by formalin and paraffin embedded for histopathological and immunohistochemistry (IHC) staining.

### 2.8. Biochemical Measurements

Whole blood samples of mice were collected by intracardiac puncture. The blood samples were centrifuged at 2000× *g* for 20 min and were separated from the serum. Biochemistry tests included creatinine and blood urea nitrogen (BUN) levels.

### 2.9. Histopathological and Immunohistochemical Analysis

The kidneys were fixed in 10% formalin, dehydrated, and embedded in paraffin. Paraffin-embedded kidney tissue sections were dried and rehydrated. The slides were incubated in 3% hydrogen peroxide for 20 min and then were placed in a microwave oven for 15 min in citrate buffer. Tissue sections were stained with hematoxylin and eosin (H+E) for histopathological analysis. For IHC staining, the slides were incubated for 2 h at room temperature with anti-cleaved-caspase 3 (Cell Signaling Technology, Ipswich, MA, USA), anti-α-SMA (Abcam, Cambridge, MA, USA) or anti-LC3 (MBL, Nagoya, Japan) antibodies. The slides were added with a secondary antibody for 1 h and were displayed using a STARR TREK Universal HRP detection kit (Biocare Medical, Concord, CA, USA). Finally, the slides were stained using hematoxylin.

### 2.10. Masson Staining

Masson trichrome staining was performed according to the protocol (ScyTek Lab., Logan, UT, USA).

### 2.11. Statistical Analysis

The results are presented as the mean ± standard deviation (SD) between groups using a one-way analysis of variance (ANOVA) followed by a post-hoc Bonferroni test or two-sample *t*-test. In all statistical tests, differences were considered significant at *p* < 0.05.

## 3. Results

### 3.1. High Levels of LTF Expression in the Kidney Tissues of AKI and CKD Patients

**Figure 1:**
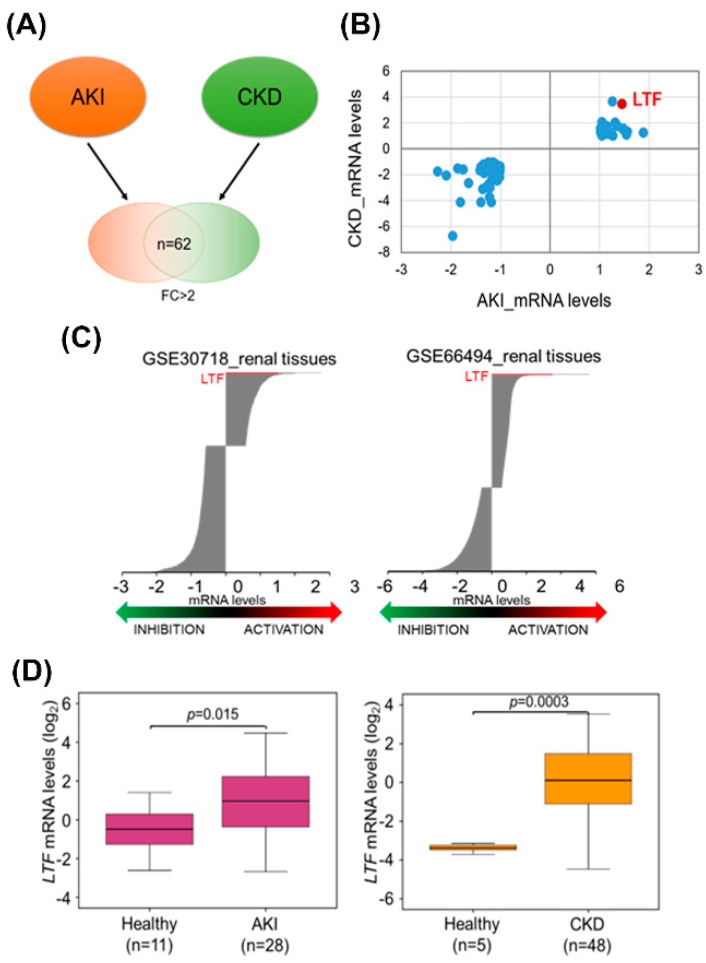
Upregulation of *LTF* in the kidney tissues of acute kidney injury (AKI) and chronic kidney disease (CKD) patients. (**A**) Expression microarray analysis determined 62 overlay genes at a threshold of two-fold change (FC) in the kidney tissues of AKI and CKD patients. (**B**) The dotplot for the mRNA levels (log_2_) of the 62 consensus genes were shown. (**C**) The mRNA levels in the kidney tissues of AKI (GSE30718) and CKD (GSE66494) patients at a threshold of 1.5-FC. (**D**) The mRNA levels of *LTF* were upregulated in the kidney tissues of AKI and CKD patients.

We first analyzed the transcriptional profiles using the microarray dataset. We identified 62 overlay genes with two-fold changes (FC) in clinical kidney tissues from AKI and CKD patients (Figure 1A and Table S1). Among the 62 overlay genes, the mRNA levels of *LTF* were notably upregulated in the kidney tissues of AKI and CKD patients (Figure 1B). All genes with 1.5 FC in kidney tissues from AKI and CKD patients are shown in Figure 1C. The results indicated that *LTF* mRNA levels were significantly increased in the renal tissues of both AKI and CKD patients (Figure 1D).

### 3.2. Lactoferrin Induces Autophagy through the Activation of AMPK and Inhibition of Akt/mTOR Pathway

**Figure 2:**
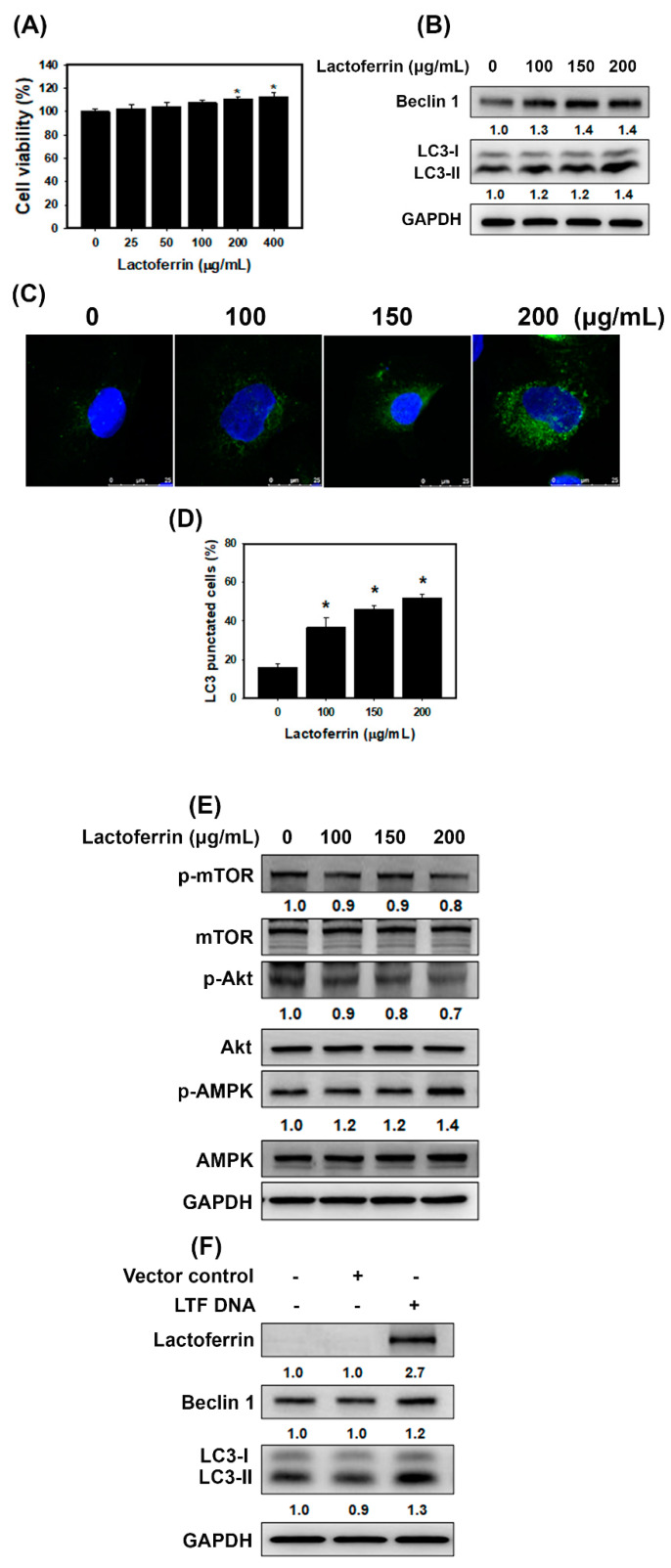
Lactoferrin induces autophagy in HK-2 cells. (**A**) Cell viability of lactoferrin-treated cells. Cells were treated with various concentrations of lactoferrin for 24 h. * *p* < 0.05 compared with control. (**B**) The protein levels of LC3 and beclin 1 in HK-2 cells treated with lactoferrin for 24 h. (**C**) Confocal immunofluorescence microscopy imaging of LC3 following 24 h treatment with lactoferrin. (**D**) Quantification of punctate LC3 staining. Cells were treated with various concentrations of lactoferrin for 24 h. * *p* < 0.05 compared with control. (**E**) Protein levels of Akt/mTOR and AMPK pathways. Cells were treated with various concentrations of lactoferrin for 24 h. (**F**) Western blotting for lactoferrin, LC3 and beclin 1 proteins derived from HK-2 cells without (vector control) or with *LTF* overexpression for 24 h.

To determine whether lactoferrin affects cell viability in HK-2 cells (a human kidney proximal tubular epithelial cell line), the cells were treated with lactoferrin at the indicated concentrations to analyze cell viability (Figure 2A). The results showed that lactoferrin did not cause obvious changes in cell viability. The high concentrations (200 μg/mL and 400 μg/mL) of lactoferrin slightly increased the viability of HK-2 cells. Furthermore, we examined whether lactoferrin induced autophagy by measuring autophagy-associated proteins using western blot analysis (Figure 2B). The levels of beclin 1 and LC3-II were markedly increased in cells treated with lactoferrin. In addition, we determined the percentage of cells with punctate LC3 staining by fluorescence microscopy (Figure 2C,D). The results found that treatment with lactoferrin caused a concentration-dependent increase in LC3 dots in HK-2 cells. A previous study reported that autophagy induction can be regulated by the activation of AMPK and inhibition of Akt/mTOR pathway [22]. As shown in Figure 2E, lactoferrin increased AMPK phosphorylation and inhibited Akt and mTOR phosphorylation. The overexpression of *LTF* could predominantly increase lactoferrin expression (Figure 2F). Furthermore, forced expression of the exogenous *LTF* gene elevated beclin 1 and LC3-II expression. These findings indicate that lactoferrin induces autophagy through the activation of AMPK and inhibition of Akt/mTOR pathway.

### 3.3. Lactoferrin Suppresses Oxidative Stress-Induced Cell Death and Apoptosis by Augmenting Autophagy in HK-2 Cells

**Figure 3:**
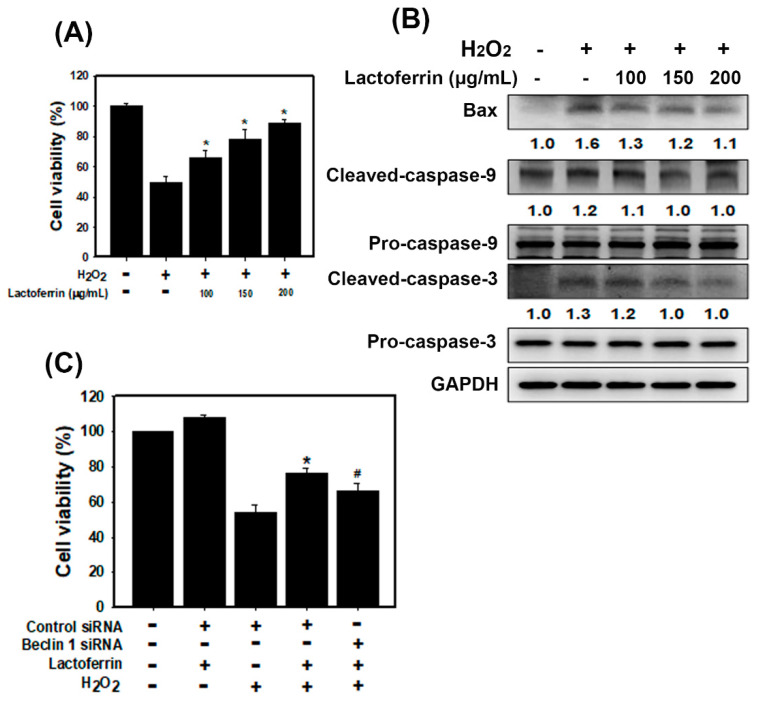
Lactoferrin reduces H_2_O_2_-induced apoptosis through the induction of autophagy in HK-2 cells. (**A**) Effect of lactoferrin on cell viability of HK-2 cells exposed to H_2_O_2_. Cells were pretreated with lactoferrin for 6 h and then treated with 500 μM H_2_O_2_ for 18 h. * *p* < 0.05 compared with H_2_O_2_ alone. (**B**) Western blot analysis of apoptosis-associated protein expression in HK-2 cells. Cells were pretreated with lactoferrin for 6 h and then treated with 500 μM H_2_O_2_ for 18 h. (**C**) Cell viability is shown in the absence or presence of Beclin 1 siRNA. Cells were transfected with Beclin 1 siRNA for 24 h. Then, cells were pretreated with lactoferrin for 6 h and incubated with 500 μM H_2_O_2_ for 18 h. * *p* < 0.05 control siRNA + lactoferrin + H_2_O_2_ compared with control siRNA + H_2_O_2_. # *p* < 0.05 control siRNA+ lactoferrin + H_2_O_2_ compared with Beclin 1 siRNA + lactoferrin + H_2_O_2_.

Hydrogen peroxide (H_2_O_2_) is an oxidant of inflammation that contributes to the pathogenesis of chronic diseases [23]. To address whether lactoferrin attenuates oxidative stress-induced damage, we analyzed the effect of lactoferrin on H_2_O_2_-induced damage by analyzing cell viability (Figure 3A). The results showed that lactoferrin substantially inhibited H_2_O_2_-induced cell death in HK-2 cells. Moreover, H_2_O_2_ increased apoptosis-related protein expression, including cleaved caspase-9, cleaved caspase-3, and bax (Figure 3B). Pretreatment with lactoferrin suppressed H_2_O_2_-induced apoptosis in HK-2 cells. To further validate the role of lactoferrin-promoted autophagy in H_2_O_2_-induced cell death, a beclin 1-targeting siRNA was designed and transfected into HK-2 cells. As shown in Figure 3C, beclin 1 siRNA-transfected cells displayed significantly decreased viability compared to that of cells transfected with control siRNA. These results suggest that lactoferrin inhibits oxidative stress-induced cell death and apoptosis, by augmenting autophagy.

### 3.4. Lactoferrin Has an Antifibrotic Role in Human Kidney Proximal Tubular Cells

**Figure 4:**
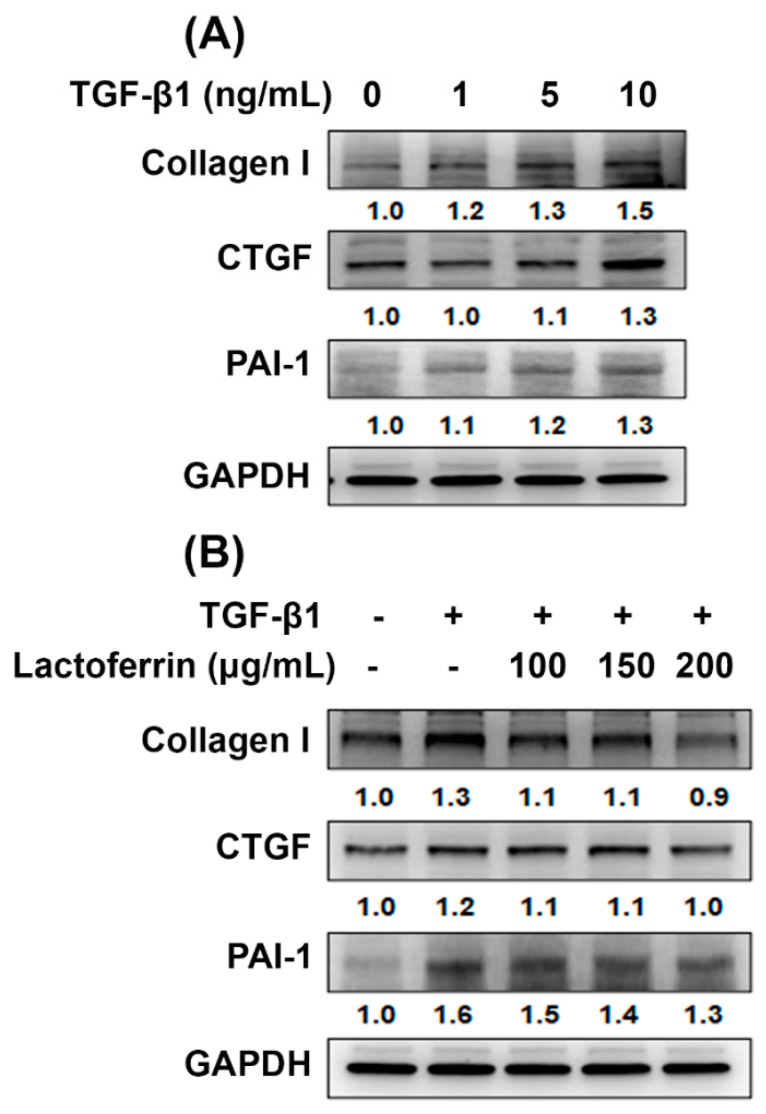
Lactoferrin inhibits TGF-β1-induced fibrosis in HK-2 cells. (**A**) TGF-β1 increases fibrosis-related protein, including collagen 1, CTGF and PAI-1. Cell were incubated with TGF-β1 for 24 h. (**B**) Effect of lactoferrin on fibrosis-related proteins expression in HK-2 cells exposed to TGF-β1 (10 ng/mL). Cells were pretreated with lactoferrin for 24 h and then incubated with TGF-β1 for 24 h.

The excessive matrix proteins, accumulation of fibroblasts and loss of functioning nephrons are main pathological characteristic of progressive CKD and cause renal fibrosis [24]. Transforming growth factor-β1 (TGF-β1) is a significant mediator in renal fibrosis [24,25]. The connective tissue growth factor (CTGF) and plasminogen activator inhibitor-1 (PAI-1) are known to be potent inducers of tissue fibrosis [26,27]. We found that TGF-β1 increased the expression of CTGF, PAI-1 and collagen 1 in a concentration-dependent manner (Figure 4A). Next, we stimulated cultured renal epithelial cells (HK-2) with TGF-β1 in the presence or absence of lactoferrin, to analyze whether lactoferrin inhibits the TGF-β1-induced fibrosis signaling pathway. The results showed that lactoferrin reduced the expression of the profibrogenic TGF-β1 target genes PAI-1, CTGF and collagen I (Figure 4B). The results indicate that lactoferrin inhibits TGF-β1-induced renal fibrosis.

### 3.5. Folic Acid (FA) Induces AKI and Develops Early Fibrosis in Kidney Tissues

**Figure 5:**
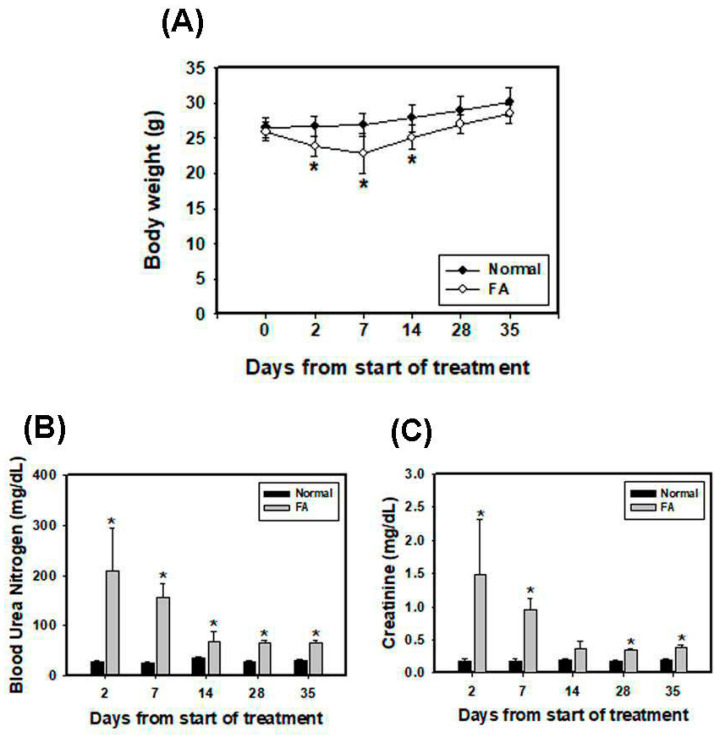

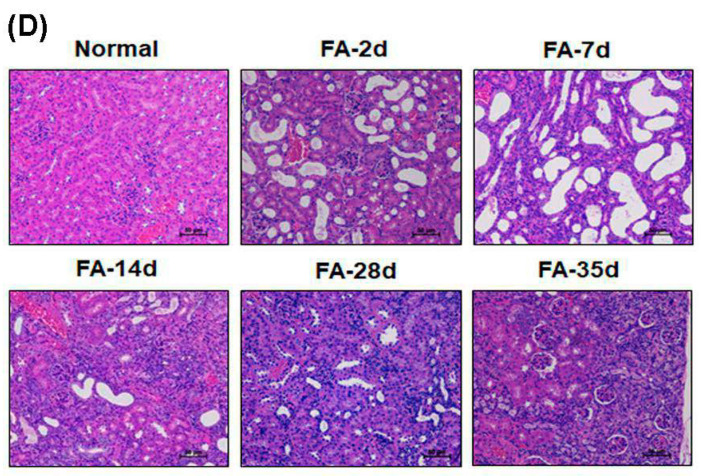
Folic acid (FA) induces AKI and renal fibrosis development. (**A**) Measurement of body weights of C57BL/6 mice. Mice were i.p. injected with 250 mg/kg FA one time at day 0. * *p* < 0.05 compared with normal. Renal function was assessed at 2, 7, 14, 28 and 35 days postinjection by measuring blood urea nitrogen (BUN) (**B**) and creatinine (**C**) levels. * *p* < 0.05 compared with normal. (**D**) Representative kidney sections in mice were stained with H+E and examined by microscopy. Scale bar = 50 μm.

Ischemia/reperfusion (I/R), sepsis, toxicants and drugs are usual causes of AKI and induce tubular necrosis and reduce glomerular filtration [28]. Previous studies have demonstrated that high doses of folic acid (FA) cause AKI due to the formation of luminal crystals and toxicity in the tubular epithelium in rodents [29,30]. FA-induced AKI can cause CKD and fibrosis in a mouse model [31]. As shown in Figure 5A, mice given 250 mg/kg FA intraperitoneally had decreased body weight at days 2, 7 and 14 and recovered at days 28 and 35. BUN concentrations and serum creatinine levels predominantly increased after FA injection at days 2 and 7 and decreased until day 14 (Figure 5B,C). Renal histopathology was examined using H+E staining at days 2, 7, 14, 28 and 35 (Figure 5D). The results showed that the renal cortex after FA treatment displayed brush border loss and tubular dilatation at days 2 and 7. At day 14, there was a partial recovery of normal renal structure characterized by repopulation of the tubular epithelial cells, but brush border restoration showed that it was incomplete. FA caused marked glomerular atrophy, which is a hallmark of CKD, on day 35. Furthermore, renal fibrosis was assessed by Masson’s trichrome staining for collagen fibers (Figure S1). The results showed the significant accumulation of collagen fibers in the FA group at days 28 and 35. In summary, peak renal dysfunction, including serum creatinine and BUN, occurred 2 days after FA administration and gradually decreased at day 14. Although the two markers recovered, the persistent damage of normal tubular morphology was examined in kidneys until day 35, revealing incomplete recovery and early CKD progression.

### 3.6. Lactoferrin Is a Therapeutic Intervention in the AKI to CKD Continuum

**Figure 6:**
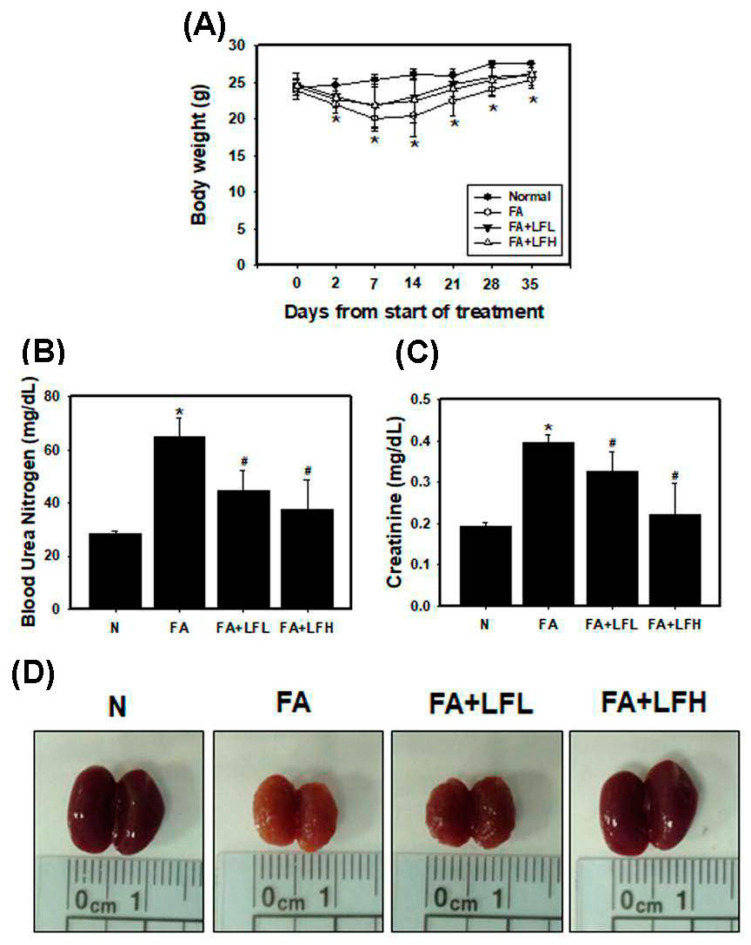
Renoprotective effects of lactoferrin on the FA mouse model. (**A**) Measurement of body weights of C57BL/6 mice. * *p* < 0.05 compared with FA. Renal function was assessed by measuring BUN (**B**) and creatinine (**C**) levels. * *p* < 0.05 compared with normal. * *p* < 0.05 compared with normal. # *p* < 0.05 compared with FA. (**D**) Direct observations of kidneys from the mice.

**Figure 7:**
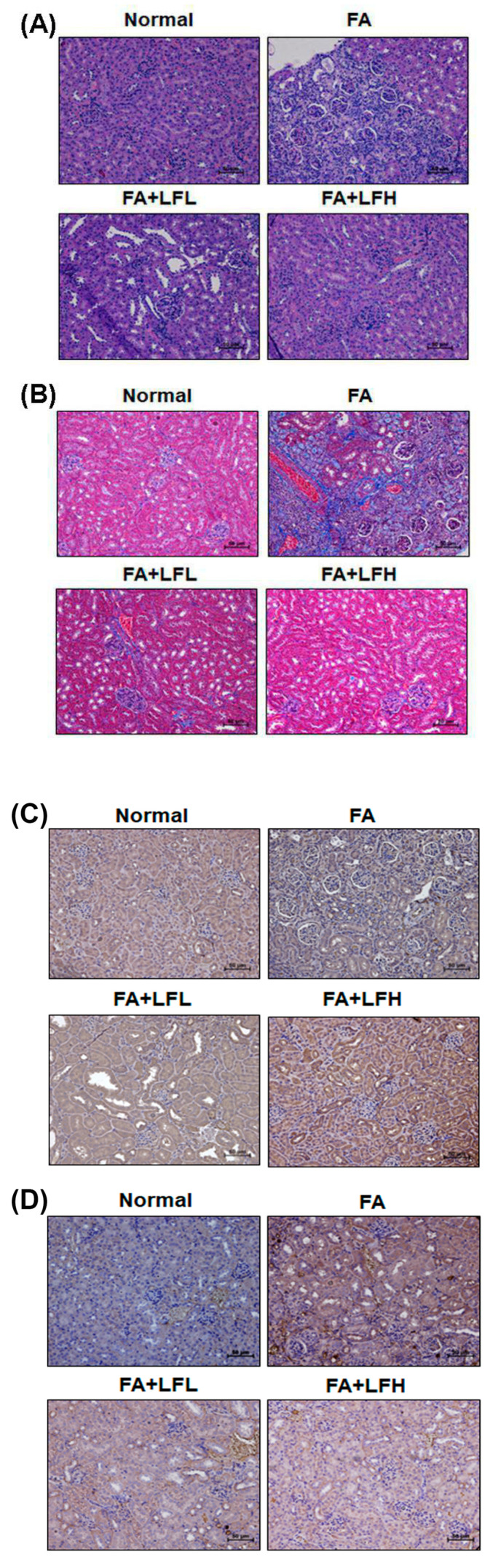
Lactoferrin attenuated renal injury and renal fibrosis in FA-induced mice. Representative micrographs of H+E (**A**) and Masson’s trichrome staining (**B**) in the indicated groups in the FA mouse model. The protein expression of LC3 (**C**) and cleaved caspase-3 (**D**) in the indicated groups of kidney sections. Scale bar = 50 μm.

To confirm whether lactoferrin had renoprotective effects on FA-induced AKI to CKD transition, we examined renal function, body weight and the appearance of kidneys. The results indicated that FA injection decreased body weight, but low and high concentrations of lactoferrin (LFL and LFH) restored body weight (Figure 6A). FA decreased renal function as creatinine and BUN levels increased compared to those of the normal group (Figure 6B,C). LFL and LFH can inhibit FA-induced creatinine and BUN levels. Furthermore, kidneys from the mice were directly examined (Figure 6D). We found that the kidneys of the FA group were orange in color and that the surfaces of the kidneys were rough and uneven. The lactoferrin groups (FA + LFL and FA + LFH) had improved appearances. In addition, the kidney sections of the mice in the FA-treated groups showed variable and severe histopathological alterations (interstitial inflammation and glomerular atrophy) (Figure 7A). Normal histological structures with normal renal tubules and glomeruli were observed in the FA + LFH group. The assessment of renal fibrosis in kidneys by Masson’s trichrome staining and immunohistochemical staining for profibrotic marker (α-SMA) showed significant renal fibrosis development in the FA group (Figure 7B and Figure S2). Fibrosis was restrained in LF-treated mice in comparison to that in FA-treated mice. Moreover, to analyze whether the lactoferrin-induced renoprotection was indeed due to the regulation of autophagy and apoptosis, we examined the markers of autophagy and apoptosis. The results showed that the FA + LFH group had markedly increased LC3 expression (Figure 7C). The protein levels of cleaved caspase-3 were highly elevated after FA injection, whereas they were significantly suppressed in the FA + LFL and FA + LFH groups (Figure 7D). Thus, treatment with lactoferrin recovered renal function and further suppressed renal fibrosis through the inhibition of apoptosis and the induction of autophagy in the AKI to CKD continuum.

## 4. Discussion

AKI is an important contributor to the increasing risk of developing CKD [3,32]. Among the findings to date, irregular repair of AKI may cause CKD through excessive deposition of components of the extracellular matrix, cell death, persistent inflammation and fibrosis [32,33]. However, the underlying molecular mechanisms in the AKI to CKD continuum are unclear. In the current study, we analyzed the transcriptional profiles using the microarray dataset in clinical kidney tissues from AKI and CKD patients (Figure 1). The results found that the mRNA level of LTF was significantly upregulated in the kidney tissues of AKI and CKD patients (Figure 1B–D). A previous study found the protective effect of lactoferrin on cisplatin-induced nephrotoxicity in rats. Furthermore, lactoferrin ameliorates cisplatin-induced creatinine and BUN in plasma [34]. Lactoferrin protects the kidney against chromium-induced AKI through antioxidative, antiproliferative and anti-inflammatory effects by the downregulation of IGF-1 and IL-18 [20]. In addition, lactoferrin inhibits oxidative stress, apoptosis and neuroinflammation through the upregulation of brain-derived neurotrophic factor (BDNF) and hypoxia-inducible factor 1α (HIF-1α) and the inhibition of JNK and P38 in neurons [35]. Our results showed that lactoferrin suppressed oxidative stress-induced cell death and apoptosis in human kidney tubular epithelial cells (Figure 3A,B). Moreover, lactoferrin inhibited TGF-β1-induced renal fibrosis by restraining the expression of the profibrogenic genes CTGF, PAI-1 and collagen I (Figure 4B). In an in vivo study, lactoferrin ameliorated FA-decreased body weights and inhibited FA-induced creatinine and BUN levels. (Figure 6A–C). In addition, lactoferrin restored FA-induced histopathological alterations in the kidney sections of the mice (Figure 7A). Fibrosis and apoptosis were attenuated in lactoferrin-treated mice in comparison to fibrosis and apoptosis in FA-treated mice (Figure 7B,D).

A previous study found that lactoferrin induces autophagy via low-density lipoprotein receptor-related protein 1 and AMP-activated protein kinase activation [36]. Autophagy enhances cell survival and maintains cellular and tissue homeostasis [37]. Current evidence suggests that abnormal autophagy has been implicated in many human diseases, including various kidney diseases (diabetic nephropathies, AKI, polycystic kidney diseases and CKD) [38,39,40]. Autophagy dysfunction can cause a loss of podocytes, glomerulosclerosis and damage to proximal tubular cells. Autophagy also restrained apoptosis of mesangial cells in diabetic nephropathy, through the inhibition of the TGF-1 and PI3K/Akt pathways [41]. In addition, the LC3B knockout mice showed autophagy deficiency and had severe tubulointerstitial fibrosis after ureter obstruction [42]. Therefore, the protective mechanisms of autophagy help repair and regenerate damaged kidneys [40]. In our current study, lactoferrin induced autophagy via the activation of AMPK and the inhibition of the Akt/mTOR pathway in HK-2 cells (Figure 2). The folic acid mouse model showed that the kidney sections of lactoferrin-treated mice markedly increased autophagy (Figure 7C). The knockdown of autophagy significantly accelerated H_2_O_2_-induced cell death (Figure 3C). These results suggest that lactoferrin inhibits oxidative stress-induced cell death and apoptosis by augmenting autophagy.

## 5. Conclusions

**Figure 8:**
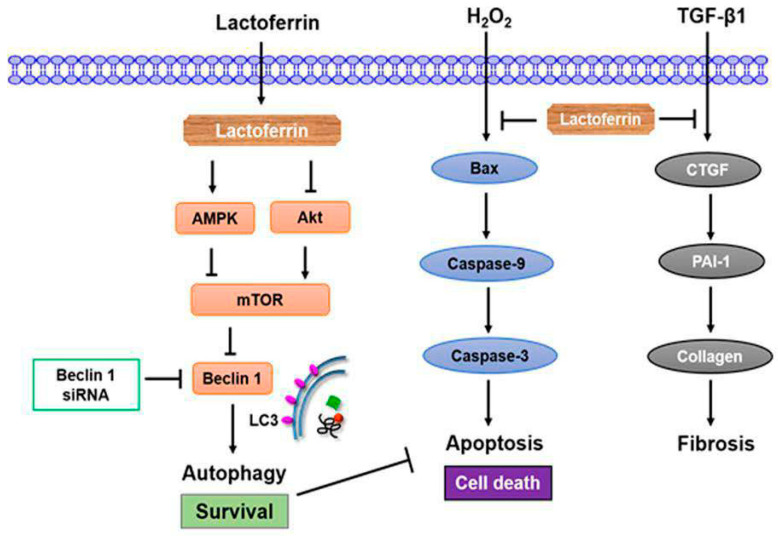
Lactoferrin contributes the renoprotective effect in the AKI to CKD transition. Lactoferrin induces autophagy through the activation of AMPK and inhibition of Akt/mTOR pathway in human kidney tubular epithelial cells. Furthermore, lactoferrin suppresses oxidative stress-induced apoptosis by augmenting autophagy. Lactoferrin restrains renal fibrosis. Therefore, lactoferrin is an effective therapeutic intervention for the AKI to CKD continuum.

In summary, high levels of LTF expression were observed in AKI and CKD patients. Lactoferrin induced autophagy through the activation of AMPK and the inhibition of Akt/mTOR pathway in human kidney tubular epithelial cells (Figure 8). Lactoferrin suppressed oxidative stress-induced cell death and apoptosis by augmenting autophagy. Furthermore, lactoferrin has an anti-fibrotic role in human kidney cells. In an in vivo study, lactoferrin was an effective therapeutic intervention in the AKI to CKD continuum.

## Supplementary Materials

The following are available online at https://www.mdpi.com/1999-4923/12/5/434/s1, Figure S1: FA induces renal fibrosis. Representative kidney sections in mice were stained with Masson’s trichrome staining and examined by microscopy. Scale bar = 60 μm; Figure S2: Immunohistochemistry (IHC) of fibrotic marker protein α-SMA in kidney of FA mouse model. The protein expression of α-SMA in the indicated groups of kidney sections. Scale bar = 60 μm; Table S1: The identification of 62 overlay genes with 2-fold changes in the kidney tissues of AKI and CKD patients.

## References

[1] Fang Y., Teng J., Ding X. (2015). Acute kidney injury in China. Hemodial. Int..

[2] Horne K.L., Packington R., Monaghan J., Reilly T., Selby N.M. (2017). Three-year outcomes after acute kidney injury: Results of a prospective parallel group cohort study. BMJ Open.

[3] Coca S.G., Singanamala S., Parikh C.R. (2012). Chronic kidney disease after acute kidney injury: A systematic review and meta-analysis. Kidney Int..

[4] Hingorani S., Molitoris B.A., Himmelfarb J. (2009). Ironing out the pathogenesis of acute kidney injury. Am. J Kidney Dis..

[5] Chen Y., Lin L., Tao X., Song Y., Cui J., Wan J. (2019). The role of podocyte damage in the etiology of ischemia-reperfusion acute kidney injury and post-injury fibrosis. BMC Nephrol..

[6] Stallons L.J., Whitaker R.M., Schnellmann R.G. (2014). Suppressed mitochondrial biogenesis in folic acid-induced acute kidney injury and early fibrosis. Toxicol. Lett..

[7] He L., Wei Q., Liu J., Yi M., Liu Y., Liu H., Sun L., Peng Y., Liu F., Venkatachalam M.A. (2017). AKI on CKD: Heightened injury, suppressed repair, and the underlying mechanisms. Kidney Int..

[8] Bostanciklioglu M. (2019). An update on the interactions between Alzheimer’s disease, autophagy and inflammation. Gene.

[9] Camuzard O., Santucci-Darmanin S., Carle G.F., Pierrefite-Carle V. (2019). Role of autophagy in osteosarcoma. J. Bone Oncol..

[10] Jiang S., Sun J., Mohammadtursun N., Hu Z., Li Q., Zhao Z., Zhang H., Dong J. (2019). Dual role of autophagy/mitophagy in chronic obstructive pulmonary disease. Pulm. Pharmacol. Ther..

[11] Li L., Kang H., Zhang Q., D’Agati V.D., Al-Awqati Q., Lin F. (2019). FoxO3 activation in hypoxic tubules prevents chronic kidney disease. J. Clin. Invest..

[12] Peng X., Wang Y., Li H., Fan J., Shen J., Yu X., Zhou Y., Mao H. (2019). ATG5-mediated autophagy suppresses NF-kappaB signaling to limit epithelial inflammatory response to kidney injury. Cell Death Dis..

[13] Li H., Peng X., Wang Y., Cao S., Xiong L., Fan J., Wang Y., Zhuang S., Yu X., Mao H. (2016). Atg5-mediated autophagy deficiency in proximal tubules promotes cell cycle G2/M arrest and renal fibrosis. Autophagy.

[14] Lin Y.F., Lee Y.H., Hsu Y.H., Chen Y.J., Lin Y.F., Cheng F.Y., Chiu H.W. (2017). Resveratrol-loaded nanoparticles conjugated with kidney injury molecule-1 as a drug delivery system for potential use in chronic kidney disease. Nanomedicine.

[15] Zhang Y., Wang L., Meng L., Cao G., Wu Y. (2019). Sirtuin 6 overexpression relieves sepsis-induced acute kidney injury by promoting autophagy. Cell Cycle.

[16] Levay P.F., Viljoen M. (1995). Lactoferrin: A general review. Haematologica.

[17] Berlutti F., Pantanella F., Natalizi T., Frioni A., Paesano R., Polimeni A., Valenti P. (2011). Antiviral properties of lactoferrin--a natural immunity molecule. Molecules.

[18] Velusamy S.K., Poojary R., Ardeshna R., Alabdulmohsen W., Fine D.H., Velliyagounder K. (2014). Protective effects of human lactoferrin during Aggregatibacter actinomycetemcomitans-induced bacteremia in lactoferrin-deficient mice. Antimicrob. Agents Chemother..

[19] Tsubota A., Yoshikawa T., Nariai K., Mitsunaga M., Yumoto Y., Fukushima K., Hoshina S., Fujise K. (2008). Bovine lactoferrin potently inhibits liver mitochondrial 8-OHdG levels and retrieves hepatic OGG1 activities in Long-Evans Cinnamon rats. J. Hepatol..

[20] Hegazy R., Salama A., Mansour D., Hassan A. (2016). Renoprotective Effect of Lactoferrin against Chromium-Induced Acute Kidney Injury in Rats: Involvement of IL-18 and IGF-1 Inhibition. PLoS ONE.

[21] Abrink M., Larsson E., Gobl A., Hellman L. (2000). Expression of lactoferrin in the kidney: Implications for innate immunity and iron metabolism. Kidney Int..

[22] Bort A., Sanchez B.G., Mateos-Gomez P.A., Diaz-Laviada I., Rodriguez-Henche N. (2019). Capsaicin Targets Lipogenesis in HepG2 Cells Through AMPK Activation, AKT Inhibition and PPARs Regulation. Int. J. Mol. Sci..

[23] Goldkorn T., Balaban N., Shannon M., Chea V., Matsukuma K., Gilchrist D., Wang H., Chan C. (1998). H_2_O_2_ acts on cellular membranes to generate ceramide signaling and initiate apoptosis in tracheobronchial epithelial cells. J. Cell Sci..

[24] Eddy A.A., Neilson E.G. (2006). Chronic kidney disease progression. J. Am. Soc. Nephrol..

[25] Bottinger E.P. (2007). TGF-beta in renal injury and disease. Semin. Nephrol..

[26] Grotendorst G.R., Okochi H., Hayashi N. (1996). A novel transforming growth factor beta response element controls the expression of the connective tissue growth factor gene. Cell Growth Differ..

[27] Eddy A.A., Fogo A.B. (2006). Plasminogen activator inhibitor-1 in chronic kidney disease: Evidence and mechanisms of action. J. Am. Soc. Nephrol..

[28] Thadhani R., Pascual M., Bonventre J.V. (1996). Acute renal failure. New Engl. J. Med..

[29] Long D.A., Price K.L., Ioffe E., Gannon C.M., Gnudi L., White K.E., Yancopoulos G.D., Rudge J.S., Woolf A.S. (2008). Angiopoietin-1 therapy enhances fibrosis and inflammation following folic acid-induced acute renal injury. Kidney Int..

[30] Fink M., Henry M., Tange J.D. (1987). Experimental folic acid nephropathy. Pathology.

[31] Leelahavanichkul A., Yan Q., Hu X., Eisner C., Huang Y., Chen R., Mizel D., Zhou H., Wright E.C., Kopp J.B. (2010). Angiotensin II overcomes strain-dependent resistance of rapid CKD progression in a new remnant kidney mouse model. Kidney Int..

[32] Zuk A., Bonventre J.V. (2019). Recent advances in acute kidney injury and its consequences and impact on chronic kidney disease. Curr. Opin. Nephrol. Hypertens..

[33] Bonventre J.V., Yang L. (2011). Cellular pathophysiology of ischemic acute kidney injury. J. Clin. Invest..

[34] Kimoto Y., Nishinohara M., Sugiyama A., Haruna A., Takeuchi T. (2013). Protective effect of lactoferrin on Cisplatin-induced nephrotoxicity in rats. J. Vet. Med. Sci..

[35] Xu S.F., Zhang Y.H., Wang S., Pang Z.Q., Fan Y.G., Li J.Y., Wang Z.Y., Guo C. (2019). Lactoferrin ameliorates dopaminergic neurodegeneration and motor deficits in MPTP-treated mice. Redox Biol..

[36] Aizawa S., Hoki M., Yamamuro Y. (2017). Lactoferrin promotes autophagy via AMP-activated protein kinase activation through low-density lipoprotein receptor-related protein 1. Biochem. Biophys. Res. Commun..

[37] Mizushima N. (2007). Autophagy: Process and function. Genes Dev..

[38] Levine B., Kroemer G. (2008). Autophagy in the pathogenesis of disease. Cell.

[39] Mizushima N., Levine B., Cuervo A.M., Klionsky D.J. (2008). Autophagy fights disease through cellular self-digestion. Nature.

[40] Lin T.A., Wu V.C., Wang C.Y. (2019). Autophagy in Chronic Kidney Diseases. Cells.

[41] Ding Y., Choi M.E. (2015). Autophagy in diabetic nephropathy. J. Endocrinol..

[42] Ding Y., Kim S., Lee S.Y., Koo J.K., Wang Z., Choi M.E. (2014). Autophagy regulates TGF-beta expression and suppresses kidney fibrosis induced by unilateral ureteral obstruction. J. Am. Soc. Nephrol..

