# Supplementary material for: Lactoferrin Contributes a Renoprotective Effect in Acute Kidney Injury and Early Renal Fibrosis
Source: Pharmaceutics. 2020 May 8;12(5):434. doi: 10.3390/pharmaceutics12050434 (PMC7284869; doi:10.3390/pharmaceutics12050434)
Supplement: Supplementary file 1 [file pharmaceutics-12-00434-s001.pdf]

pharmaceutics

Supplementary Materials: Lactoferrin Contributes a Renoprotective Effect in Acute Kidney Injury and Renal Fibrosis

Yung-Ho Hsu, I-Jen Chiu, Yuh-Feng Lin, Yi-Jie Chen, Yu-Hsuan Lee and Hui-Wen Chiu

Table S1. The identification of 62 overlay genes with 2-fold changes in the kidney tissues of AKI and CKD patients.

ADAM9	C16orf89	C2orf54	C7	CDH6	CFI	CHI3L1
COL14A1	COPA	CR2	DPT	EGF	ELF5	ESRRB
FBXL21	FGB	FTCD	GAD1	GALM	GDF15	GGT6
HAVCR1	HOXB8	HSP90B1	ITLN1	KLK1	KNG1	LCN2
LGSN	LINC00937	LPP	LTF	LYZ	MET	MFAP5
MT1E	MT1F	MT1M	NNMT	OGN	OLFM4	OSMR
PAPPA	PTN	PVALB	REN	SEMA6D	SGK494	SLC12A3
SLC22A7	SLC23A3	SLC7A13	SOST	SOX9	STAT1	TEF
TMEM106A	TPCN2	TRPM3	TRPM6	USP2	VCAM1	

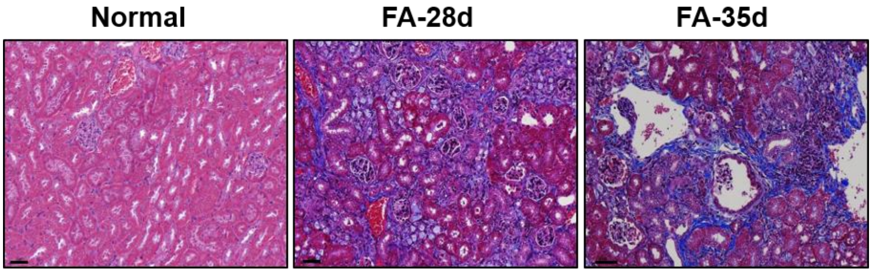

Figure S1. FA induces renal fibrosis. Representative kidney sections in mice were stained with Masson's trichrome staining and examined by microscopy. Scale bar = 60 μm.

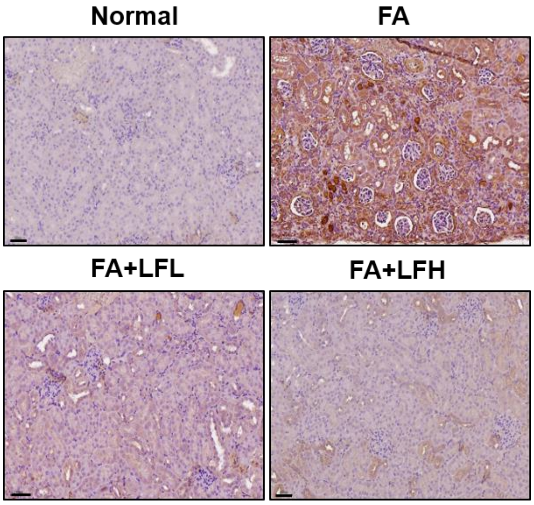

Figure S2. Immunohistochemistry (IHC) of fibrotic marker protein α-SMA in kidney of FA mouse model. The protein expression of α-SMA in the indicated groups of kidney sections. Scale bar = 60 μm.
